# Postcraniotomy Function of the Temporal Muscle in Skull Base Surgery: Technical Note Based on a Preliminary Study

**DOI:** 10.1100/2012/427081

**Published:** 2012-03-12

**Authors:** Amr Abdulazim, Andreas Filis, Pooyan Sadr-Eshkevari, Fried Schulte, Nora Sandu, Bernhard Schaller

**Affiliations:** ^1^Department of Neuroanatomy and Molecular Brain Research, Ruhr-University, Bochum, Germany; ^2^Department of Neurosurgery, University of Erlangen-Nürnberg, Erlangen, Germany; ^3^Farzan Clinical Research Institute, Tehran, Iran; ^4^Department of Neurosurgery, University of Münster, Münster, Germany; ^5^Department of Neurosurgery, University of Paris, Paris, France; ^6^University of Oradea, Oradea, Romania

## Abstract

*Purpose*. Patients undergoing craniotomies necessitating preparation of the temporal muscle (TM) may experience postoperative functional impairment of the temporomandibular joint. This topic has not been thoroughly discussed in the literature so far. In the present study, the authors propose a questionnaire as an evaluation tool to assess to what degree different TM preparation techniques correlate with postoperative temporomandibular joint dysfunction. *Materials and Methods*. Between 2004 and 2006, 286 patients underwent either pterional or temporal craniotomies in the department of craniotomies at the University of Münster in Germany. Intraoperatively the TM was prepared either interfascial, submuscular, or subfascial. A patient-based questionnaire was designed and validated (Kendalls-*τ* = +1) in order to evaluate the patients' postoperative temporomandibular functional outcome. Based on strict inclusion/exclusion criteria, 69 patients were eligible for the application of the questionnaire in this preliminary study. *Results*. Seventeen percent of the patients complained of either temporomandibular joint pain (3%) or restricted mouth opening (13%) postoperatively in a follow-up period between 3 and 12 months. In 92% postoperative complaints were reported within the first 3 months and in 58% of the patients with complaints the pain eased off. In 34% a therapy was required for the pain to be controlled. In one patient (8%) a postoperative arthroscopy has been necessary. Of the patients who experienced postoperative complaints, 67% had undergone temporal and 33% pterional craniotomy. In the group where postoperatively there were issues of temporomandibular pain/dysfunction, 42% had had the TM dissected, in 25% incised, and in 8% transected. For 25% of the patients, the type of intraoperative manipulation remained unknown. *Conclusion*. For postoperative quality control, the questionnaire showed to be a suitable evaluation tool. Concerning the different preparation techniques, subfascial preparation of the TM tends to result in less postoperative complaints and is thus recommended.

## 1. Introduction

Pterional and temporal craniotomies are amongst the most common and frequently used approaches in neurosurgery. In both, the temporal muscle (TM) has to be mobilized. However, postoperative TM dysfunction including temporomandibular joint pain and mastication impairment (e.g., due to muscle atrophy as a result of direct injury, ischemia, inadequate muscle tension, or muscle denervation) remain serious complications [1]. In the neurosurgical literature there are no comparable studies dealing with postoperative TM function, comparing different surgical techniques of TM mobilization. This may be due to the fact that these patients are postoperatively referred to dentists or oral and maxillofacial surgeons than to neurosurgeons. Few data are available on postoperative mastication dysfunction in different surgical procedures with a maximum follow-up of up to 6 months [2, 3]. Therefore, the aim of our study was to design and validate a questionnaire as an evaluation tool for patients with postoperative temporomandibular joint pain and mastication dysfunction. In our preliminary retrospective study, 69 of 286 patients who underwent either pterional or temporal craniotomies between 2004 and 2006 were contactable. The questionnaire was applied to those 69 patients with a follow-up of up to 12 months.

## 2. Materials and Methods

### 2.1. Patient Population

We retrospectively reviewed the case histories of 286 patients who underwent either temporal or pterional craniotomies between 2004 and 2006 at the Department of Neurosurgery of the University Hospital of Münster in Germany.

Inclusion criteria for our study were age between 15 and 85 years and pterional or temporal craniotomies. Excluded were those patients, who had died by the time of evaluation, and those who were otherwise unavailable for evaluation. Thus, 69 patients were included in the study of which 34 (48%) were men and 35 (52%) were women, with age range between 46 and 60 years for women and between 46 and 75 years for men. The underlying disease for craniotomy in these 69 patients was cerebral tumor (39%), cerebral aneurysm (25%), space occupying intracerebral hematoma (16%), traumatic brain injury (13%), ischemic infarction of the territory of the middle cerebral artery (1%), and others (6%). Preoperative examination included medical history, physical examination, clinical laboratory diagnostics, computerized tomography (CT) or magnetic resonance imaging (MRI) of the head, as well as digital subtraction angiography (DSA) or MR-angiography in some cases.

### 2.2. Surgical Technique and Intra- and Perioperative Management

Operations were performed according to standardized surgical and anesthesiological procedures. Pterional and temporal craniotomies have been performed as described previously [4, 5]. The mobilization of the TM was performed via either dissection, transsection, or incision.

### 2.3. Different Techniques Used for Temporal Muscle Dissection

#### 2.3.1. Interfascial

This technique is extensively described by Yasargil at the beginning of the 1980s [5]. The superficial lamina of the temporalis fascia is incised from the most anterior part of the inferior temporal line as far as the root of the zygoma and then reflected anteriorly with the scalp, exposing the superficial fat pad between the two laminae. The anterior quarter of the TM is still covered by the deeper lamina of the temporal fascia, which is incised and dissected from the frontozygomatic process and zygoma.

#### 2.3.2. Submuscular

When the superficial layer of the temporal fascia comes into view, the TM is incised and dissected free from the temporal bone, leaving the temporal fascia with its fat pads in situ covering the muscle. The dissection should be performed following the direction of the fibers to avoid tearing the muscle from the deeper part of the temporal fossa, close to the zygoma, in the direction of the superior temporal line. During this maneuver, we avoid the use of monopolar coagulation.

#### 2.3.3. Subfascial

This technique allows wide exposure of the zygoma and full TM mobilization. The incision over the temporal fascia is performed close to the temporal line and parallel to the skin incision in a semilunar fashion and includes both superficial and deep laminae. The dissection of the temporal fascia must reach the superior border of the zygoma and the frontozygomatic suture. The deep layer is incised along the medial aspect of the zygoma. The TM is stripped from the infratemporal fossa using the same retrograde technique as above. The TM is then incised, leaving a cuff of fascia at the superior temporal line, for reapproximation or totally mobilizing the muscle free from the bone.

### 2.4. Anesthetic Technique

Patients fasted for at least 8 hours and were orally premedicated with midazolam prior to surgery. Routine monitoring during surgery included electrocardiography, heart rate, noninvasive mean arterial blood pressure (MABP), pulse oxymetry, esophageal temperature, end-tidal concentration of oxygen (O_2_), carbon dioxide (CO_2_), and desflurane. Except for noninvasive MABP (every 3 minutes), each parameter was measured constantly during the surgical procedure. Anesthesia was induced with intravenous (i.v.) propofol (2 mg/kg) and sufentanil (1 *μ*g/kg) followed by relaxation with rocuronium (0.6 mg/kg) i.v. prior to endotracheal intubation. Patients were now mechanically ventilated with an inspiratory oxygen concentration of FiO_2_ = 0.4. Anesthesia was maintained by a constant i.v. application of sufentanil (1 *μ*g/kg) and desflurane 6%. When necessary an additional i.v. bolus of sufentanil was applied.

### 2.5. Questionnaire

To our knowledge there is no questionnaire in the neurosurgical literature assessing systematic evaluation of postcraniotomy complications and complaints according to TM dysfunction. Here, a questionnaire has been designed to assess the occurrence of postoperative functional disorders, time of occurrence, if additional therapy has been necessary and if the latter led to improvement (see the Appendix). Finally, preexisting condition and comorbidities that might have influenced the TM dysfunction and the postoperative recovery were recorded. The questionnaire comprised 5 items:

Item A: patients complaints that seemed to be associated with the operation,
Item B: time after surgery when patients reported complaints,
Item C: whether these complaints led the patient to seek medical advice and intervention,
Item D: time range in which a relief from postoperative complaints set in and if this relief set in spontaneously or after medical intervention,
Item E: preexisting disorders and comorbidities.

Before the questionnaire was applied in the preliminary study, a validation had been necessary. On that account patients (*n* = 12) for whom the postoperative course was known from the patients' dossiers were contacted and the questionnaire was applied in a pretest fashion to allow a conclusion on the concurrent criterion-related validity. Validation was made by a board-certified neurosurgeon with help of the existing patient's charts. Furthermore, to test the internal consistency of the items in our questionnaire the value “Kendalls-*τ*” that is considered to be a measure for test reliability was calculated [6]. In terms of the construct validity it is necessary to prove that the applied items are correlated with the predicted postoperative mastication dysfunction using Spearman's correlation coefficient. The same patients were reinterviewed one month after the primary interview to obtain the test-retest reliability. These results showed a satisfactory quality of the questionnaire to assess the postoperative outcome of TM after skull base surgery, good validity, and a high clinical relevance.

### 2.6. Statistical Analysis

Data are presented as the mean ± SD. For statistical comparison of the data different statistical tests were used. For quantitative data the *t*-test was used. Qualitative data were tested either using the *χ* ^2^-test or for small samples using Fischer's exact test. The level of significance was set at a probability value of *α* < 0.05.

## 3. Results

### 3.1. Patients

Sixty-nine patients met the inclusion criteria. 48% (*n* = 34) were men and 52% (*n* = 35) women with an age range between 46 and 75 for men and between 46 and 60 for women. Underlying pathologies could be divided into three main categories: (i) cerebrovascular lesions (42%; *n* = 29), (ii) cerebral tumors (39%; *n* = 27), and (iii) trauma (13%; *n* = 9). Six percent (*n* = 4) did not fit into one of the latter three subgroups. All operations were performed by experienced senior consultants.

Among the tumor subgroup temporal craniotomy had been performed in 56% and pterional in 44%. In the subgroup with cerebrovascular lesions pterional craniotomy was used in 55% and temporal craniotomy in 41%. In 4% of these patients a craniectomy was necessary. In posttraumatic patients a temporal approach was implemented in 78% and pterional approach in 22% of the patients. Among the other 6% of patients temporal craniotomy (75%) was more often used than a pterional craniotomy (25%). Overall, temporal craniotomy was used in 54% (*n* = 37), pterional craniotomy in 45% (*n* = 31), and a craniectomy in 1% (*n* = 1).

As for the preparation of the TM, submuscular preparation was performed in 29% (*n* = 20), subfascial preparation in 15% (*n* = 10), and an interfascial preparation in 12% (*n* = 8) of the cases. In 44% (*n* = 31), however, the intraoperative preparation technique remained unknown and was, therefore, retrospectively not evaluable. There was no correlation between different craniotomy approaches and the different TM preparation technique.

### 3.2. Validation of the Questionnaire

Regarding the assessment of the internal consistency of the designed questionnaire, each of the established items revealed a Kendalls-*τ* value of +1. Thus, each item proved to be sufficiently adequate in terms of internal consistency. The analysis of the test-retest revealed no significant differences of the patients' responses to items. Therefore, the criterion of the test-retest reliability is fulfilled. Checking the construct validity showed, that the scores obtained from the assessment of each item were significantly correlated to the assessment of the global question of postoperative mastication dysfunction (Spearman's correlation coefficient; *ρ* = 1). Sufficient construct validity is thus proved.

### 3.3. Postoperative Complications

Of the 69 patients, 17% (*n* = 12) reported postoperative complaints. These were restricted and painful mouth opening in 75% (*n* = 9) and temporomandibular joint pain in 25% (*n* = 3). These patients were clinically reevaluated by oral and maxillofacial surgeons coming up with the conclusion that the cause of the pain by opening of the mouth has been shortening of the TM due to scarring of the muscle tissue in all 9 patients. In 45% (*n* = 4) of these patients the TM has been prepared submuscularly, interfascially in 22% (*n* = 2), and subfascially in 11%. In 22% (*n* = 2) the intraoperative TM preparation technique was unknown. Among the patients with temporomandibular joint pain, limited remodeling of the joint seemed to be the cause in 67% (*n* = 2) and arthrotic degeneration in 33% (*n* = 1) as shown in examinations by oral and maxillofacial surgeon. There was no correlation between the incidence of postoperative pain and the urgency of the operation (emergency versus electivity).

### 3.4. Time of Occurrence of Postoperative Complaints

Among the 12 patients with postoperative complaints, 92% (*n* = 11) reported to have experienced the complaints within the first three postoperative months. Only 1 patient (8%) reported the occurrence of the complaints after completion of the first three months postoperatively but within the first 6 months.

### 3.5. Therapy of Postoperative Complaints

Fifty-eight percent (*n* = 7) of the patients with postoperative complaints did not seek additional medical advice or therapy. However, in 29% (*n* = 2), spontaneous relief was reported within the first 6 postoperative months. In 71% (*n* = 5) postoperative complaints persisted for more than a year, but except for one patient all symptoms diminished over time. Forty-two percent (*n* = 5) of the patients consulted a physician and sought intervention. Of these, 40% (*n* = 2) received a splint therapy from a dentist. Another 40% (*n* = 2) were referred to physiotherapy. In both groups the therapy has been successful. One patient of the treated group (20%) has been appointed for arthroscopy (not yet performed at the time of manuscript submission).

### 3.6. Underlying Pathology

Patients with postoperative complaints showed different underlying pathologies that lead to operative intervention and craniotomy. In 58% (*n* = 7) an underlying intracranial tumor was the cause for surgical intervention. 25% (*n* = 3) suffered from a space occupying hematoma and in 17% (*n* = 2) the underlying disease was an aneurysm. Among the 5 patients that required therapy for the postoperative complaints the underlying disease was a tumor in 60% (*n* = 3) and a hematoma in 40% (*n* = 2).

### 3.7. Surgical Approach and Preparation of the Temporalis Muscle

The different craniotomies and TM preparation techniques were further considered in relation to the occurrence of postoperative complaints. Of symptomatic patients (*n* = 12), 67% (*n* = 8) and 33% (*n* = 4) had temporal and pterional craniotomies, respectively. Regarding the TM preparation, it was submuscular in 42% (*n* = 5), interfascial in 25% (*n* = 3), and subfascial in 8% (*n* = 1). The preparation technique of the TM was unknown in 25% (*n* = 3). Both the hypotheses that temporal craniotomy (*P* = 0.221) and more invasive preparation of the TM (*P* = 0.543) predispose to more and prolonged postoperative TM complaints were not significant.

## 4. Discussion

Temporal and pterional approaches for craniotomy are amongst those most commonly used for neurosurgery. In both approaches the TM is mobilized, requiring to be refixed on bone. Postoperative pain at the temporomandibular joint and/or dysfunction seem to be common yet underreported in the literature [1, 3, 7–14]. In 2007, Rocha-Filho et al. [3] have evaluated 71 patients after pterional craniotomy for aneurysm surgery 4 to 6 months postoperatively in view of postcraniotomy complications. They report that 48% of the patients complained of pain during dental evaluation and that 28% were bothered by pain during normal jaw movement.

In the present study, we have designed a questionnaire for the systematic evaluation of postcraniotomy TM dysfunctions in neurosurgical patients. This is not only the way for postoperative quality evaluation but also a better standard in the research of postcraniotomy pain. Our present study is a follow-up project of earlier studies after retrosigmoidal removal of vestibular schwannoma [10, 11]. Our follow-up was up to 12 months—in contrast to a maximum of 6 months in previous studies [3, 8].

Patients were evaluated retrospectively and all the information was obtained from patients' personal reports. Nevertheless, given correct validation and reliability, as in our study (Spearman's correlation coefficient, *ρ* = 1 and Kendall's-*τ* = +1), such questionnaires are considered well established [9]. For that reason, the patients' questionnaire represents a valuable instrument for postoperative quality evaluation. However, it must be emphasized that it is difficult to depict the patients' complaints in a consistent scoring system when such assessment is necessarily subjective. Additionally, due to a missing medical documentation/detection of postoperative complaints, only the existence or nonexistence of temporomandibular joint pain and mastication dysfunction were considered as valuation standards, as these are easily evaluable by retrospective interrogation. From the recent work of Hwang et al. [15], it seems, for example, that TM atrophy, by comparing volumetric measurements of the TM on the ipsi- and contralateral side, cannot only be demonstrated but also quantified in postoperative magnetic resonance imaging studies.

In a study by Kawaguchi et al. [8], postcraniotomy complaints occurred within the first the 3 months postoperatively in complaint patients (20%). Our study showed postcraniotomy complaints in 17% of the reevaluated patients. Except for one patient the postoperative complaints occurred within the 3 months, in accordance with the results of Kawaguchi et al. [8]. Patients reported of spontaneous relief of limited mouth opening in a similar way as has been shown by Kawaguchi et al. [8]. Kawaguchi et al. [8] has concentrated solely on the applied craniotomy as a cause of different functional outcome. In contrast to this, our own experience seems to reveal that different preparation techniques of the TM with respect to invasivity have more influence on postcraniotomy TM function than the performed craniotomy. Theoretically, other factors like urgency of the operation, experience of the surgeon, or choice of the surgical approach could also influence outcome. Surprisingly, not much attention has been made on such factors in previous neurosurgical literature. Oikawa et al. [16], for example, give detailed advice to prevent TM atrophy in pterional craniotomy, but there are no data for the preferred inferior-to-superior-dissection of the TM compared with other surgical techniques. We showed, however, that in patients with postoperative complaints the more invasive dissection had been performed in 42%. Less invasive techniques have been incision in 25% and transection in 8%. There was however a tendency in patients whom a less aggressive dissection was applied to have less postoperative dysfunction of the temporal muscle. The fact that these results were not statistically significant may on the one hand be attributable to our small patient numbers, but invasivity of TM preparation may not be the only factor to be considered. This needs to be taken into consideration in upcoming prospective studies on larger patient collectives.

## 5. Conclusion

We were able to create a valuable patient-based questionnaire which can be used as a tool for postoperative TM assessment. This represents a new standard in the postoperative quality control. The study demonstrates a tendency that less aggressive and less invasive preparation and dissection of the TM causes less postoperative dysfunction. In our opinion it is important to conduct controlled randomized studies with larger patient numbers to raise the profile of a topic which seems to be not thoroughly discussed in the neurosurgical literature.

## Appendix

Questionnaire for evaluation of postoperative function of the temporalis muscle after skull base surgery.

Item A: After the neurosurgical operation, do you had any difficulties in
  - Chewing: *yes-no *
  - Bitting: *yes-no *
  - Opening mouth: *yes-no *
  - Closing mouth: *yes-no *
Item B: If you have answered *yes *in any of the questions of (1), please differentiate when the problems occurred:
  - Immediately after operation: *yes-no *
  - Within the first 2 weeks after operation: *yes-no *
  - More than 2 weeks after operation: *yes-no *
  - In the long-term follow-up: *yes-no *
  - If you have answered *yes* in any of the questions of (1), please differentiate if you have pain/problems until now: *yes-no *
Item C: If you have still problems, as stated in (2), what was done in the past:
  - By the dentist?
  - If *yes*: Please specify.
  - By a (neuro) surgeon?
  - If *yes*: Please specify
  - Reoperation required: *yes-no *
  - Others
Item D: Was the treatment as indicated in (3) successful?
*Yes-no*
If *yes*: you are pain-free now? *yes-no *
If *no*: the pain persists until now? *yes-no *
Item E: We have some additional question to your patient's history: Do you have previously any
  - rheumatological disorders? *yes-no *
  - Osteoporosis? *yes-no *
  - Diabetes mellitus? *yes-no *
  - Immunsuppression and/or transplantation?   *yes-no *
  - Allergy? *yes-no *
  - Vascular pathologies, arteriosclerosis, heart attack? *yes-no *
  - Radiation of tumors? *yes-no *
  - Others? *yes-no *
